# Disparities in tobacco use by adolescents in southeast, Nigeria using Global Youth Tobacco Survey (GYTS) approach

**DOI:** 10.1186/s12889-018-5231-1

**Published:** 2018-03-06

**Authors:** Ijeoma U. Itanyi, Chika N. Onwasigwe, Scott McIntosh, Tamara Bruno, Deborah Ossip, Emmanuel A. Nwobi, Chima A. Onoka, Echezona E. Ezeanolue

**Affiliations:** 1 Department of Community Medicine, University of Nigeria Teaching Hospital, Enugu, Nigeria; 2 Department of Community Medicine, Faculty of Medical Sciences, University of Nigeria, Ituku-Ozalla Campus, Enugu, Nigeria; 3 Department of Public Health Sciences, University of Rochester, Rochester, NY USA; 4 School of Community Health Sciences, University of Nevada, Las Vegas, USA

**Keywords:** Adolescent, Tobacco, GYTS, Nigeria, Urban, Rural, Smoking, Cigarettes

## Abstract

**Background:**

Tobacco use during adolescence is a substantial problem and adolescents are at higher risk of addiction and prolonged use. To reduce the burden of tobacco-related morbidity and mortality, monitoring of adolescent tobacco use is imperative. We aimed to determine the prevalence of tobacco use among adolescents in urban and rural secondary schools in Enugu State, southeast Nigeria.

**Methods:**

A cross-sectional study of 4332 adolescents in 8th to 10th grades in 25 urban and 24 rural secondary schools in Enugu, Nigeria was done using Global Youth Tobacco Survey (GYTS) methodology. Students were asked about previous and current tobacco use, smoking cessation, and susceptibility to smoking initiation among non-smokers. Geographical, age and sex prevalence differences were examined. Analyses were performed for all adolescents (10–19 years) and for a subset of students, 13–15 years of age for comparison with previous GYTS surveys. All analyses were weighted to account for the complex survey design and for differential non-response at school, class and student levels.

**Results:**

About 28.9% of students reported ever smoking cigarettes; 19.4% reported current tobacco use among all adolescents (13.3, 5.8 and 7.8% for cigarettes, other smoked tobacco, and smokeless tobacco, respectively) while 18.6% reported current tobacco use among 13–15 year olds (12.6, 5.2 and 7.5% for cigarettes, other smoked tobacco and smokeless tobacco respectively). Prevalence of all types of tobacco use was higher in rural schools (vs. urban schools), and among boys (vs. girls). Susceptibility to smoking initiation among non-smokers was 9.3% (95% CI: 8.1–10.7) among all adolescents, and 9% (95% CI: 7.6–10.7) among 13–15 year olds. About 88.1% of all adolescent smokers desired to quit and 57.9% of them had never received help to quit smoking.

**Conclusions:**

Nearly one in every five school-going adolescents currently uses at least one type of tobacco in Enugu State, southeast Nigeria. Prevalence of tobacco use is higher in rural schools and among boys in this setting. Most adolescent current smokers desire to quit and need smoking cessation support.

**Electronic supplementary material:**

The online version of this article (10.1186/s12889-018-5231-1) contains supplementary material, which is available to authorized users.

## Background

Tobacco smoking is a major cause of preventable global mortality, resulting in about 5 million deaths annually [1, 2]. The death toll is projected to reach 10 million by 2020 [3]. In addition, an estimated 600,000 people die from exposure to second-hand smoke annually [4], with 75% of these deaths among women and children [5]. The adverse health effects of tobacco use have been well established [6], and in total, if unabated, the tobacco epidemic is expected to cause one billion deaths in this century [7]. Effective tobacco control measures are crucial to prevent this public health crisis.

Adolescents, ages 10 to 19 years, are particularly vulnerable to high-risk behaviours like tobacco use [8]. Tobacco use during adolescence increases the risk of addiction [9, 10], and most regular adult smokers became addicted to nicotine during adolescence [11, 12, 13]. The tobacco industry specifically targets youth, especially in developing countries, to retain them as lifetime users [14] who will experience prolonged exposure to tobacco and its associated health risks.

Globally, tobacco use is a substantial problem among adolescents. One in three school adolescents has ever smoked cigarettes worldwide, with about 25% of them smoking their first cigarette before the age of 10 [15]. Almost 20% of secondary school students were reported to currently use tobacco worldwide, and almost 10% were current smokers [16]. African studies have shown the prevalence of tobacco smoking among school-going adolescents to range from 2.5% in Malawi [17] to 27% in rural Zambia [18]. The use of adolescent smokeless tobacco was also found to be high (18%) in the Democratic Republic of Congo [19].

In Nigeria, adolescent tobacco use is also a significant problem. The 2008 Global Youth Tobacco Survey (GYTS) found that one in five students aged 13 to 15 years had ever experimented with cigarette smoking, and about one in ten students currently smoked cigarettes [20]. Studies in other parts of Nigeria showed tobacco smoking prevalence among school-going adolescents to range from 3.4% in Ibadan in the south-west [21] to 34.8% in Akwa Ibom State in the south-south [22].

Most studies on adolescent tobacco use in Nigeria have been in urban areas, leaving a gap in knowledge of the magnitude of the problem in rural areas. Such studies also lacked rigorous and robust methodology. Two studies in 2001 and 2008, respectively, implemented the typically comprehensive GYTS methodology but they did not include the southeast and northeast regions of Nigeria [20, 23, 24]. Moreover, data from the last GYTS study in 2008 may not reflect the current problem of adolescent tobacco use in Nigeria. Two previous studies in southeast Nigeria [25, 26] were conducted in only public schools so prevalence of adolescent tobacco use in private schools remains unknown. There is also a paucity of studies that have compared urban and rural adolescent tobacco use in Nigeria.

The objective of this study was to determine the prevalence of tobacco use among adolescents in urban and rural secondary schools in Enugu, southeast Nigeria. Our research questions were: 1) What is the prevalence of tobacco use among adolescents in urban and rural secondary schools in Enugu State, southeast Nigeria? 2) Are there geographical differences in prevalence of tobacco use among adolescents? 3) Are there sex differences in prevalence of tobacco use among adolescents? 4) Are there age differences in prevalence of tobacco use among adolescents?

To the best of our knowledge, this is the first comprehensive study to examine disparities in the prevalence of tobacco use by adolescents in urban and rural secondary schools in Nigeria.

## Methods

### Study area

The study area, Enugu State, is one of five states in southeast region of Nigeria. It has 17 Local Government Areas (LGAs), administrative units of the state, five of which are urban and 12 are rural. Enugu State has 242 urban secondary schools and 393 rural secondary schools, of which 283 are public schools while 352 are private schools [27].

### Study design

A cross-sectional study design was used to compare findings from school-based surveys of two groups of schools, defined by their urban or rural location (or strata) within Enugu State. A school survey was used because it is easy to administer, relatively inexpensive, provides reliable results and refusal is significantly lower than in household surveys. Also, some adolescents may not wish to discuss a potentially sensitive topic like tobacco use in their homes.

Different approaches for studying adolescent tobacco use exist, but the GYTS design was chosen because it is comprehensive and collects data on all key tobacco control indicators used to monitor youth tobacco use. It also uses a standardised methodology for the study procedures to enable comparison between studies. Some minor modifications were made where appropriate to fit the Nigerian setting. For example, country-specific values were included in the options to some questions in the questionnaires.

### Study population

The study population comprised adolescents in 8th to 10th grades (Junior Secondary 2 and 3 [JS2 and JS3], and Senior Secondary 1 [SS1]), in both public and private secondary schools in Enugu State. Students were included in the study if they were in 8th, 9th or 10th grade in selected schools. All students in selected classes were eligible to participate in the survey. Students in the selected classes who were absent on the day of data collection or who refused to participate were excluded from the survey.

### Sample size

The minimum sample size required for the study was computed using the formula for determination of sample size for comparison of independent proportions [28]. Using power of 90%, significance level of 0.05, prevalence of 26.1% from previous GYTS in Nigeria [23], prevalence difference of 5%, and response rate of 80%, the minimum sample size calculated was 1890 students per group. This corresponded with the minimum sample size of 1875 students from 25 schools required for studies using GYTS methodology [29]. A sample of 2000 students from 25 schools in each study group was sought, resulting in a total of 4000 students from 50 schools for the entire study; 80 students per school.

### Sampling technique

All secondary schools in Enugu State were identified and stratified into urban and rural using the host LGAs. The 2014/2015 session school enrolment list, obtained from the Ministry of Education, contained the most current student enrolment information by grade and gender. This was modified by creating a separate enrolment list for each stratum. Two-stage cluster sampling was used to select the students independently within each stratum, with the probability of selection proportionate to school enrolment size.

In the first stage, 25 schools were selected from each stratum using systematic random sampling. The schools were the primary sampling units. In each stratum, the schools in the sampling frame were sorted in descending order by enrolment size. The cumulative population of eligible students in each school was calculated. To determine the sampling interval (SI), the total number of eligible students was divided by 2000 - the sample size for each stratum. A number, designated random start (RS), was chosen randomly between 1 and the SI. The school within which the RS fell was selected first, after which subsequent schools were selected by adding the SI to the RS. Selection of schools used the formula: RS; RS + SI; RS + 2SI; RS + 3SI, etc. Schools for which the cumulative population contained one of the serial numbers calculated from the series above were selected [29]. Microsoft Office Excel 2013 was used for sampling at this stage. Schools with a population size of less than 80 were excluded from each sampling frame before the 1st stage of sampling. The proportions of students excluded from the urban and rural sampling frames were 5.6 and 10.7% respectively. The urban sampling frame had 172 schools, with a population size of 60,601, while the rural sampling frame had 285 schools and a population size of 59,173.

**Fig. 1:**
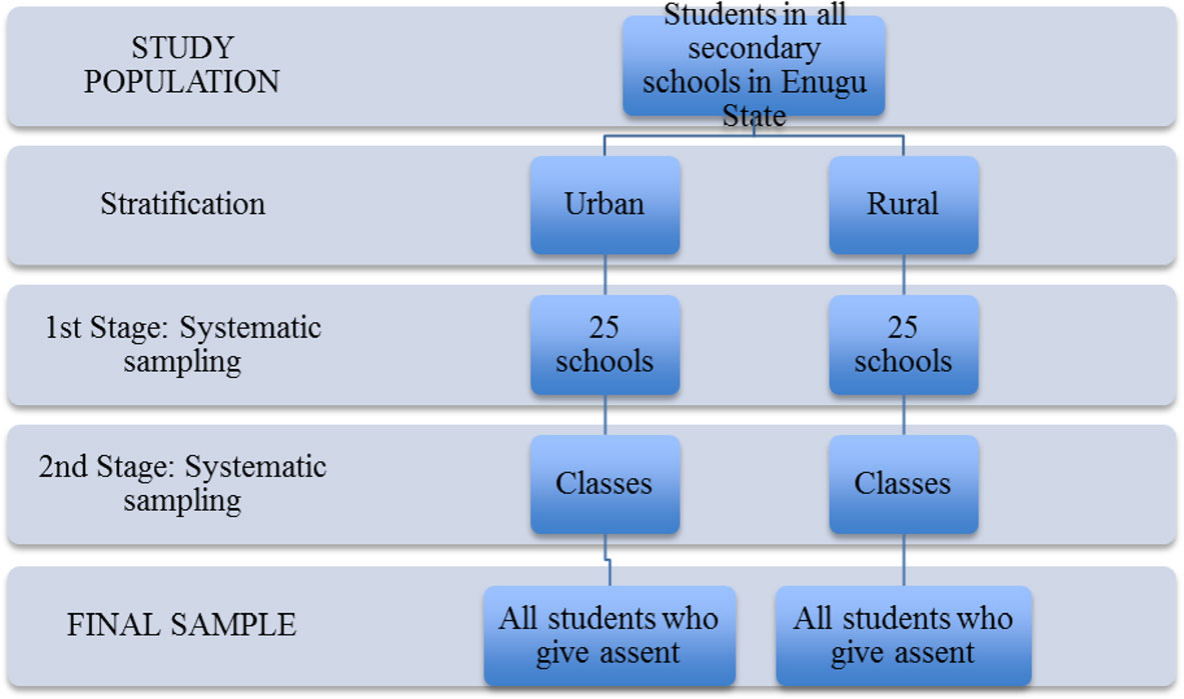
Flow Diagram of Sampling Technique

In the second stage, classes were selected from each school using systematic random sampling. All students in the selected classes who were present on the day of survey administration were eligible to participate. There was no sample substitution for schools or classes that refused to participate in the survey because this would have altered the representativeness of the sample and thus would have led to biased results. Every effort was made to ensure the cooperation of selected schools and classes. (See Fig. 1 for a flow diagram of the sampling).

### Data collection

An anonymous, self-administered, semi-structured questionnaire adapted from the GYTS Core Questionnaire with optional questions Version 1.1 [30] was used to collect information about students’ socio-demographic characteristics, use of smoked and smokeless tobacco, and susceptibility to smoking initiation (Additional file 1). The questionnaire was first pre-tested with 162 students in three secondary schools that were not selected for the study: two urban (one public and one private) and one rural. These schools were located at least ten kilometres from the schools that were selected for the study. After correction of ambiguities identified in the questionnaires, the survey was administered in English in November and December, 2015. Teachers were absent during survey administration to enable the students to be free to answer the questions. During survey administration, one rural private school was found to be ineligible so no data were collected from that school.

### Definition of variables

*Ever use of tobacco* (cigarettes, other smoked tobacco, and smokeless tobacco) was defined as experimentation with any of the aforementioned tobacco products. *Current use of cigarettes* was defined as use on 1 day or more within the past 30 days. *Current use of other smoked tobacco products (pipes, cigars, water pipes/shisha or bidis) or smokeless tobacco* was defined as any use within the past 30 days*. Any tobacco use* was defined as use of one or more types of tobacco products, and both ever use and current use were examined. *Dual tobacco use* was defined as current use (within past 30 days) of any two tobacco products. *Multiple tobacco use* was defined as current use (within past 30 days) of cigarettes, other smoked tobacco and smokeless tobacco. *Susceptibility to smoking initiation within the next year* was defined as the absence of a firm decision not to smoke within the next year. This was determined if a student did not answer “definitely no” to the question ‘*At any time during the next 12 months, do you think you will use any form of tobacco?’*

### Statistical analyses

Weights were initially calculated for each student record to account for the complex survey design and for differential non-response at school, class and student levels, using the GYTS approach [29]. The final weight for each record was a product of the school selection weight, class selection weight, and overall non-response adjustment factor. The school selection weight was the inverse probability of selecting a school. The class selection weight was the inverse probability of selecting a class in a school. The overall non-response adjustment factor was the product of school, class and student non-response adjustment factors. Weights were computed separately for each stratum.

Data analysis was performed using Stata Version 11. Prevalence estimates and 95% confidence intervals were computed for each type of tobacco use. Geographical (urban/rural), age and sex differences in prevalence of adolescent tobacco use were examined with Pearson Chi-square test using ‘svy’ command in Stata to account for the complex survey design. Differences in prevalence by grade and school type (public/private) were also examined. Differences in susceptibility to smoking initiation among non-smokers were examined by school geographical location, age and sex. Geographical differences in pattern of tobacco use – age at first use of cigarettes and smokeless tobacco, and number of cigarettes smoked per day – were ascertained. Smoking cessation was also examined. For all analyses, *p*-values of < 0.05 were considered statistically significant. All analyses were weighted to account for the complex survey design and adjusting for non-response.

Analyses were performed for all adolescents (ages 10–19 years), and for a subset of adolescent students (13–15 years of age) to enable comparison with previous studies.

### Ethical considerations

Ethical approval for the study was obtained from the Health Research Ethics Committee of the University of Nigeria Teaching Hospital (NHREC/05/01/2008B-F-WA00002458-1RB00002323). Approval was also obtained from the Ministry of Education and the principals of the selected schools. Written assent was obtained from the students after a detailed explanation of the study objectives, procedures, risks and benefits. Written informed consent was also obtained from principals of the selected schools, who acted as the legal guardians of the students, as was done in previous GYTS studies in Nigeria.

## Results

Of 4354 students surveyed, 4332 were adolescents (ages 10–19 years); 2230 from 25 urban schools, and 2102 from 24 rural schools. Response rates were 84.4 and 80.6% in urban and rural locations, respectively. The median age of the students was 14 years (14 years in urban schools and 15 years in rural schools) [interquartile range = 2 years]; 43.6% were boys; 28.1% were in JS 2, 36.2% in JS 3 and 35.7% in SS 1.

### Overall prevalence of tobacco use and prevalence by geographical location

#### All adolescents

Approximately three in ten students (28.9%) reported they had ever smoked cigarettes. Prevalence of reported ever experimentation with other smoked tobacco and smokeless tobacco were 11.7 and 26.8%, respectively. Reported use of tobacco within past 30 days were 13.3 for cigarettes, 5.8% for other smoked tobacco and 7.8% for smokeless tobacco. Approximately one in five students (19.4%) reported current use of at least one type of tobacco product, 5.8% reported dual use, and 1.8% reported current use of all three types of tobacco. Students in rural schools reported higher use of all types of tobacco than students in urban schools, with all but current smokeless tobacco use reaching statistical significance (Table 1).

**Table 1:** Prevalence of tobacco use among adolescents (10–19 years) in urban and rural secondary schools in Enugu State

Variable	Prevalence (95% CI)			*F*	*P* value
	All adolescents (10–19 years)				
	All (*n* = 4332)	Urban (*n* = 2230)	Rural (*n* = 2102)		
Ever use of tobacco					
Cigarettes	28.9 (25.6–32.3)	23.1 (19.9–26.6)	35.1 (29.5–41.2)	14.07	0.0005
Other smoked tobacco	11.7 (9.9–13.5)	9.3 (7.8–11.2)	14.3 (11.4–17.8)	8.81	0.0047
Smokeless tobacco	26.8 (24.1–29.5)	22.7 (20.1–25.6)	31.1 (26.6–36.0)	10.41	0.0023
Current use of tobacco					
Cigarettes	13.3 (11.1–15.6)	10.1 (8.3–12.2)	16.7 (13.0–21.2)	10.33	0.0024
Other smoked tobacco	5.8 (4.5–7.1)	4.0 (3.2–5.1)	7.6 (5.5–10.5)	10.11	0.0026
Smokeless tobacco	7.8 (6.2–9.4)	6.5 (5.3–8.0)	9.2 (6.7–12.5)	3.45	0.0694
Any kind of tobacco	19.4 (16.5–22.2)	15.7 (13.4–18.3)	23.3 (18.4–29.0)	7.94	0.0070
Both cigarettes and other smoked tobacco	3.4 (2.5–4.4)	2.1 (1.5–2.9)	4.9 (3.3–7.2)	11.95	0.0012
Both cigarettes and smokeless tobacco	3.8 (2.7–4.8)	2.6 (2.0–3.4)	4.9 (3.2–7.4)	6.69	0.0128
Both smokeless and other smoked tobacco	2.2 (1.4–3.0)	1.4 (1.0–2.1)	3.0 (1.8–5.0)	5.82	0.0198
All 3 types of tobacco	1.8 (1.1–2.5)	1.1 (0.7–1.8)	2.6 (1.5–4.4)	6.15	0.0168
	13–15 year olds				
	All (*n* = 2992)	Urban (*n* = 1587)	Rural (*n* = 1405)		
Ever use of tobacco					
Cigarettes	28.1 (24.4–31.9)	22.3 (18.9–26.1)	34.7 (28.3–41.7)	11.96	0.0012
Other smoked tobacco	10.8 (8.8–12.8)	8.8 (7.1–10.9)	13.1 (9.8–17.1)	5.02	0.0299
Smokeless tobacco	25.7 (22.5–28.9)	21.8 (18.9–25.1)	30.1 (24.6–36.2)	7.03	0.0109
Current use of tobacco					
Cigarettes	12.6 (10.2–15.0)	8.8 (7.1–11.1)	16.8 (12.8–21.8)	13.60	0.0006
Other smoked tobacco	5.2 (3.8–6.6)	3.6 (2.7–4.9)	7.1 (4.8–10.2)	7.65	0.0081
Smokeless tobacco	7.5 (5.7–9.4)	6.4 (5.1–8.1)	8.7 (5.8–12.9)	1.75	0.1920
Any kind of tobacco	18.6 (15.4–21.8)	14.7 (12.3–17.3)	23.1 (17.5–29.7)	8.15	0.0064
Both cigarettes and other smoked tobacco	2.9 (1.9–4.0)	1.7 (1.0–2.8)	4.4 (2.8–6.7)	8.60	0.0052
Both cigarettes and smokeless tobacco	3.5 (2.2–4.7)	2.4 (1.6–3.5)	4.7 (2.8–7.7)	4.64	0.0364
Both smokeless and other smoked tobacco	2.0 (1.1–2.9)	1.3 (0.8–2.2)	2.8 (1.5–5.2)	3.61	0.0638
All 3 types of tobacco	1.6 (0.8–2.4)	1.0 (0.5–1.9)	2.3 (1.2–4.6)	3.36	0.0733

*F:* Design-based χ^2^

#### 13–15 year olds

Among 13–15 year olds, cigarettes were the most commonly used tobacco product (12.5%), followed by smokeless tobacco (7.5%), then other smoked tobacco (5.2%). Prevalence estimates for this age group were higher in rural schools than urban schools for all types of tobacco (Table 1).

### Sex differences in prevalence of tobacco use

#### All adolescents

Males reported significantly higher use (both ever use and current use) of all types of tobacco than females (e.g., 22.5% vs. 16.9% for any tobacco for males and females, respectively; Table 2). The most commonly used tobacco product for both sexes was cigarettes (15.6% vs. 11.5% for males and females, respectively), followed by smokeless tobacco (9.3% vs. 6.7%), then other smoked tobacco products (7.3% vs. 4.6%). Reported current dual use of tobacco products was higher among males (7.3%) than females (4.7%), *p* = 0.002. Similarly, reported current use of all three types of tobacco was about twice as high among males compared to females (Table 2).

**Table 2:** Sex differences in prevalence of tobacco use among adolescents (10–19 years) in urban and rural secondary schools in Enugu State

Variable	Prevalence (95% CI)		*F*	*P* value
	All adolescents (10–19 years)			
	Male (*n* = 1889)	Female (*n* = 2443)		
Ever use of tobacco				
Cigarettes	32.9 (28.8–37.2)	25.9 (22.5–29.6)	13.50	0.0006
Other smoked tobacco	14.1 (11.8–16.8)	9.9 (8.0–12.1)	11.06	0.0017
Smokeless tobacco	30.4 (27.1–34.0)	24.0 (20.9–27.3)	11.58	0.0014
Current use of tobacco				
Cigarettes	15.6 (12.6–19.3)	11.5 (9.6–13.8)	8.52	0.0054
Other smoked tobacco	7.3 (5.6–9.4)	4.6 (3.5–6.1)	10.09	0.0026
Smokeless tobacco	9.3 (7.4–11.6)	6.7 (5.2–8.5)	7.08	0.0106
Any kind of tobacco	22.5 (18.7–26.8)	16.9 (14.2–20.1)	9.55	0.0034
Both cigarettes and other smoked tobacco	4.3 (3.1–6.0)	2.8 (2.0–3.9)	6.16	0.0167
Both cigarettes and smokeless tobacco	5.0 (3.6–6.9)	2.8 (2.0–3.9)	11.81	0.0012
Both smokeless and other smoked tobacco	3.0 (2.1–4.4)	1.6 (1.0–2.6)	8.50	0.0054
All 3 types of tobacco	2.5 (1.7–3.8)	1.3 (0.8–2.1)	9.75	0.0031
	13–15 year olds (*N* = 2992)			
	Male (*n* = 1241)	Female (*n* = 1751)		
Ever use of tobacco				
Cigarettes	32.1 (27.6–36.9)	25.4 (21.6–29.6)	10.62	0.0021
Other smoked tobacco	13.0 (10.6–15.8)	9.3 (7.2–11.8)	7.69	0.0079
Smokeless tobacco	29.7 (25.7–34.1)	22.9 (19.6–26.6)	11.01	0.0018
Current use of tobacco				
Cigarettes	15.1 (11.8–19.0)	10.9 (8.8–13.3)	8.36	0.0058
Other smoked tobacco	6.5 (4.7–9.0)	4.3 (3.2–5.8)	6.40	0.0148
Smokeless tobacco	9.1 (6.8–12.1)	6.4 (4.9–8.4)	6.42	0.0147
Any kind of tobacco	21.4 (17.3–26.1)	16.6 (13.7–20.1)	6.54	0.0138
Both cigarettes and other smoked tobacco	3.8 (2.4–6.0)	2.3 (1.6–3.3)	4.32	0.0431
Both cigarettes and smokeless tobacco	5.1 (3.4–7.5)	2.3 (1.6–3.5)	16.08	0.0002
Both smokeless and other smoked tobacco	2.8 (1.6–4.6)	1.4 (0.8–2.6)	4.68	0.0356
All 3 types of tobacco	2.3 (1.2–4.3)	1.1 (0.6–2.0)	5.34	0.0253

*F:* Design-based χ^2^

#### 13–15 year olds

Sex differences observed in prevalence of all types of tobacco use were similar to those observed among all adolescents. Among 13–15 year olds, reported current dual use of tobacco products among males was about twice that of females (Table 2).

### Age differences in prevalence of tobacco use

Among all adolescents, there was a general pattern of higher use among students older than 15 years, with results reaching statistical significance for all three ever use measures (Table 3).

**Table 3:** Age differences in prevalence of tobacco use among adolescents (10–19 years) in urban and rural secondary schools in Enugu State

Variable	Prevalence (95% CI)			*F*	*P* value
	10–12 years (*n* = 430)	13–15 years (*n* = 2992)	16–19 years (*n* = 910)		
Ever use of tobacco					
Cigarettes	24.6 (18.7–31.6)	28.1 (24.6–32.0)	33.5 (29.0–38.3)	3.67	0.0294
Other smoked tobacco	12.4 (8.8–17.3)	10.8 (9.0–13.0)	14.5 (11.9–17.5)	3.31	0.0420
Smokeless tobacco	23.4 (18.7–29.1)	25.7 (22.7–29.1)	31.8 (28.4–35.4)	5.66	0.0048
Current use of tobacco					
Cigarettes	12.5 (8.6–17.9)	12.6 (10.4–15.2)	16.0 (13.1–19.3)	2.53	0.0866
Other smoked tobacco	6.9 (4.4–10.8)	5.2 (4.0–6.8)	7.0 (5.1–9.6)	1.83	0.1685
Smokeless tobacco	6.1 (3.9–9.3)	7.5 (5.9–9.6)	9.5 (7.4–12.1)	2.22	0.1152
Any kind of tobacco	17.9 (13.4–23.5)	18.6 (15.6–22.0)	22.5 (19.1–26.2)	2.73	0.0707
Both cigarettes and other smoked tobacco	4.2 (2.3–7.3)	2.9 (2.1–4.2)	4.7 (3.2–7.0)	2.80	0.0740
Both cigarettes and smokeless tobacco	3.5 (2.0–6.2)	3.5 (2.4–4.9)	4.8 (3.2–7.0)	1.14	0.3230
Both smokeless and other smoked tobacco	1.9 (0.8–4.1)	2.0 (1.3–3.1)	3.1 (1.9–4.9)	1.37	0.2584
All 3 types of tobacco	1.9 (0.8–4.1)	1.6 (1.0–2.7)	2.4 (1.4–4.2)	0.89	0.4044

*F:* Design-based χ^2^

### Other differences in prevalence of tobacco use

Among all adolescents, the prevalence of both ever use and current use of all types of tobacco were similar among students in 8th to 10th grades. Students who reported ever smoking cigarettes were more in public schools compared to private schools (31.7 and 23.5% respectively; *p* = 0.03). However, no differences were found in reported use of all other types of tobacco use between students in public and private schools.

### Susceptibility to smoking initiation in the next 12 months

#### All adolescents

For all adolescents, susceptibility to smoking initiation in next 12 months among non-smokers was 9.3% (95% CI: 8.1–10.7), 7.6% in urban and 11.3% in rural schools (*p* = 0.01). No age or sex differences in susceptibility to smoking initiation were found among the students.

#### 13–15 year olds

For 13–15 year olds, susceptibility to smoking initiation in next 12 months among non-smokers was 9% (95% CI: 7.6–10.7), 7.6% in urban and 10.7% in rural schools (*p* = 0.04). No age or sex differences in susceptibility to smoking initiation were found among the students.

### Pattern of tobacco use and cessation

#### All adolescents

Overall, one in five ever smokers (19.9%) reported they smoked their first cigarette before the age of ten, 20.9% in urban and 19.2% in rural schools (*p* = 0.11). Among current smokers, 12.1% smoked more than one cigarette per day; 12.8% in urban and 11.7% in rural schools (*p* = 0.4182). Overall, 34.1% of students reported they tried their first smokeless tobacco before the age of ten; 35.4% in urban and 33.1% in rural schools (*p* = 0.01).

Nearly nine in ten current smokers (88.1%) reported they desired to quit smoking, and had tried stopping smoking in last 12 months. Almost six in ten current smokers (57.9%) reported they had never received help to stop smoking. Of students who reported they had received help to stop smoking, the most common source of help was from a friend (16%), followed by a family member (11.1%), program or professional (9.1%), and more than one group of people (6%).

#### 13–15 year olds

Overall, 15.3% of ever smokers and 32.9% of students reported they smoked their first cigarette or their first smokeless tobacco respectively before the age of ten. Among current smokers, 9.8% smoked more than one cigarette per day.

Almost nine in ten current smokers (89.1%) reported they desired to quit smoking, and had tried stopping smoking in last 12 months. Six in ten current smokers (60.8%) reported they had never received help to stop smoking. Of students who reported they had received help to stop smoking, the most common source of help was from a friend (15.4%), followed by a family member (10.7%), program or professional (8.1%), and more than one group of people (4.8%).

## Discussion

This study provides the first systematic data on tobacco use for adolescent school children in the southeast region of Nigeria. This study supports the observation that tobacco use by adolescents in Nigeria is a serious problem as more than one in four adolescents reported ever trying cigarettes or smokeless tobacco, one fifth reported smoking their first cigarette before the age of ten and almost one in five currently use at least one type of tobacco. Tobacco use was more prevalent in rural schools and among boys. More than 80% of adolescents reported a desire to quit smoking, highlighting the need for accessible cessation interventions.

### Overall prevalence among adolescents

The present study found that almost one in three adolescents had ever tried smoking cigarettes and 13% of adolescents currently smoked cigarettes. These rates are much higher than those reported in Ibadan, southwest Nigeria, where 9.4% of students reported ever smoking cigarettes and 3.4% reported current use of cigarettes [21]. These differences in prevalence may be due to under-reporting of tobacco use by the students in Ibadan because their teachers were present during data collection. Similarly, two studies of female and male students in Anambra, southeast Nigeria reported lower prevalence of current cigarette smoking, 7.7% [25] and 8.7% [26] respectively. However, these studies were conducted in only public secondary schools. Therefore, tobacco use in private schools has remained unknown. In contrast, a study of secondary school students in Akwa Ibom, south-south Nigeria reported higher prevalence of current use of tobacco (34.8%) [22], but the investigators did not report their sampling method so the study may not be replicable. Furthermore, the prevalence of dual tobacco use in this study compares with findings in Limpopo, South Africa [31] but is higher than rates reported in the US national youth tobacco survey [32].

### Overall prevalence among 13 to 15 year olds

The present study’s findings of prevalence of ever use and current use of cigarettes are similar to findings from GYTS in Greece [33], where consistent GYTS procedures were followed. Yet, the results were lower than those reported in GYTS studies in Zambia [34] where 40.1% had ever smoked cigarettes, and Democratic Republic of Congo [19] where prevalence of current use of smokeless tobacco was 18%. It is noteworthy that these two African GYTS studies had response rates (RR) of 78.3 and 57% respectively, lower than the RR of 80% expected from GYTS studies. This may have affected the precision and validity of these results. Additionally, susceptibility to smoking initiation within the next year among non-smokers reported in this study is similar to findings from GYTS studies in Pakistan [35] (12%) and Philippines [36] (10.5%), indicating a possible rise in smoking prevalence in the subsequent year.

Notably, the prevalence rates of both ever use and current use of cigarettes were higher in this study compared with the 2008 GYTS in Nigeria which reported that fewer than 20% of students had ever smoked cigarettes and fewer than 10% of students currently smoked cigarettes [20]. These results may not be generalizable to all 13 to 15 year olds in Nigeria because the study excluded the southeast and northeast regions, which have contextual differences from other regions studied. However, the higher prevalence in this study may indicate a rising prevalence of adolescent tobacco use in Nigeria since the last GYTS was conducted nine years ago.

### Geographical differences in tobacco use

Findings in the present study show that prevalence of tobacco use was higher among students in rural schools compared to urban schools for all types of tobacco products. This finding could be explained by the presence of older students in rural schools compared to urban schools since tobacco prevalence was found to increase with increasing age. It is also possible that there are contextual differences in rural and urban locations which affect adolescent tobacco use but could not be identified in the present study. Similar findings have been reported by other studies, mostly outside Africa [37–39]. The study in Osun, Nigeria [37] compared only one urban and one rural school, was inadequately powered due to small sample size, and may not be generalizable to adolescents in Nigeria. Among 13 to 15 year olds, it is remarkable that the prevalence of cigarette smoking, other smoked tobacco, dual and multiple use of tobacco, were all twice as high in rural schools compared to urban schools. This underscores the need for tobacco prevention interventions to also target adolescents in rural schools. In contrast, prevalence of smokeless tobacco use was comparable in both locations indicating that the driving factors for smokeless tobacco use may be similar in both locations. However, the finding that susceptibility to smoking initiation in the next year was higher in rural schools is of utmost public health importance since it suggests that prevalence of adolescent tobacco use will continue to be higher in rural schools in the coming year.

### Sex differences in tobacco use

The prevalence of all types of tobacco use was higher among boys compared to girls, as has been similarly reported in other studies [34, 39–44]. This is in contrast to the 2008 Nigerian GYTS report which showed no difference in prevalence of current cigarette smoking between boys and girls except in Kano [20]. It was surprising that boys reported higher use of smokeless tobacco than girls – unlike the finding in a similar study among Black South Africans [45]. The tobacco product most commonly reported by both boys and girls was cigarettes – highlighting the need for interventions that target cigarettes.

### Age and grade differences in tobacco use

The present study’s finding that the prevalence of ever use of tobacco increased with increasing age, has been reported in previous studies [26, 39, 42]. On the contrary, current use of all types of tobacco was similar among the age categories studied (10–12 years, 13–15 years, and 16–19 years), consistent with the results reported in Zaria, Nigeria [46]. A possible explanation for this finding is that younger students could have been experimenting with tobacco at the time of this study. This is of concern because early exposure to tobacco can predispose adolescents to addiction and prolonged exposure leading to development of tobacco-associated diseases later in life.

In addition, this study showed no differences in current tobacco use by students in 8th to 10th grades, contrary to findings from other studies. This unusual finding was similarly observed in urban India where 6th graders used tobacco at higher rates than 8th graders [42]. A possible explanation for this finding in the present study is that 8th graders may have been experimenting with tobacco at the time of this study. The implication is similar to that of the observed age prevalence differences.

### Cessation

An important finding in the present study is that almost 90% of current smokers desired to quit smoking and had tried to quit in the last 12 months. A similar finding was reported by the 2011 GYTS in the Philippines [36]. Unfortunately, about 60% of adolescents in the present study reported they had never received help to stop smoking, unlike in other countries. In Nigeria, although some smoking cessation support exists in some hospitals, either in the form of physician advice or counselling, or the prescription of nicotine replacement therapy or anti-depressants, it is usually not covered by health insurance. There are no smoking cessation support currently available in primary healthcare facilities or in the community where it is easier for smokers to access them [47]. In addition, no specific smoking cessation support is available for adolescents in Nigeria to the best of our knowledge. The findings of this study underscore the need for culturally tailored smoking cessation programs for adolescents. These programs could use adolescents as change agents with their peers, since the most common source of help for smoking cessation reported in this study was friends. Such interventions could use peers or role models who are fluent in the native language to target rural schools.

The present study was school-based, therefore its findings may not be generalizable to all adolescents in Enugu, Nigeria. However, a significant proportion (71.1%) of adolescents are enrolled in school in Enugu State [48]. Additionally, because only students who were present in school on the day of data collection participated in the study, the findings may have been over-estimated or under-estimated depending on whether tobacco users were more likely to be in school on that day or not. This is unlikely, however, because the students were not given prior information about the study until the arrival of the study team on the day of data collection. Another limitation, common in research, is that the survey was self-administered and students may over-report or under-report their tobacco use behaviours. Although the extent of this type of bias cannot be determined from the present study, reliability studies in the US have shown good test-retest results for similar tobacco-related questions [49].

## Conclusions

The present study’s findings indicate that about one in five school-going adolescents use tobacco, with higher prevalence in rural (vs. urban) schools and among boys (vs. girls). Results also indicate that most current smokers desire to quit, and need help. Adolescents are experimenting with tobacco at early ages, a situation which highlights the need for easily accessible and culturally appropriate tobacco prevention and cessation interventions that target adolescents early in this setting. Further research is needed to fully understand the contextual differences between urban and rural schools in this setting, which affect adolescent tobacco use.

## Additional file

Additional file 1: Adapted GYTS students’ core questionnaire. Questionnaire used to collect information about students’ socio-demographic characteristics, use of smoked and smokeless tobacco, and susceptibility to smoking initiation. (PDF 364 kb)

## Abbreviations

GYTS: Global Youth Tobacco Survey
JS: Junior Secondary
LGAs: Local Government Areas
RS: Random Start
SI: Sampling Interval
SS: Senior Secondary
