# Supplementary material for: Disparities in tobacco use by adolescents in southeast, Nigeria using Global Youth Tobacco Survey (GYTS) approach
Source: BMC Public Health. 2018 Mar 6;18:317. doi: 10.1186/s12889-018-5231-1 (PMC5840734; doi:10.1186/s12889-018-5231-1)
Supplement: Supplementary file 1 — Adapted GYTS students’ core questionnaire. Questionnaire used to collect information about students’ socio-demographic characteristics, use of smoked and smokeless tobacco, and susceptibility to smoking initiation. (PDF 364 kb) [file 12889_2018_5231_MOESM1_ESM.pdf]

STUDENTS' QUESTIONNAIRE

School ID _________

Sample School ID _________

Student ID _________

Stratum _____________

LGA _____________

School Type _____________

Introduction

Thank you for participating in this survey. Before you start, please read the following information that will help you to answer the questions.

- Some of the questions will ask about smoking **cigarettes**.
- Other questions may ask about **smoking tobacco** in general that includes cigarettes and other types of smoked tobacco products.
- Other questions may ask about using **smokeless tobacco**, which is tobacco that is not smoked, but is sniffed through the nose, held in the mouth, or chewed.
- Finally, other questions may ask about any **tobacco use** or any **tobacco products** – this includes smoking cigarettes, smoking tobacco other than cigarettes, and using smokeless tobacco.
- **Please answer all questions truthfully. There will be no punishment for your answers.**
- **Remember that your answers will be kept secret and cannot be traced to you.**
- **Also remember that you can choose not to participate in the study at any time.**

Instructions

- Please read each question carefully before answering it.
- Choose the answer that best describes what you believe and feel to be correct.
- Choose only **one** answer for each question.
- Please circle the option of the answer you have chosen.
- If you have to change your answer, don't worry, just erase it completely, without leaving marks.

The first few questions ask for some background information about yourself.

1. **How old are you? (age at your last birthday)** ________ years
2. **What is your sex?**
   a. Male
   b. Female
3. **In what grade/form are you?**
   a. JS 2
   b. JS 3
   c. SS 1
4. **Are you a day student or a boarder?**
   a. Day student
   b. Boarder
5. **During an average week, how much money do you have that you can spend on yourself, however you want?**
   a. I usually don't have any spending money
   b. 100 naira or less
   c. 101 to 200 naira
   d. 201 to 500 naira
   e. 501 to 1000 naira
   f. 1001 to 2000 naira
   g. More than 2000 naira
6. **Do your parents (or guardians) work?**
   a. Father (stepfather or guardian uncle) only
   b. Mother (stepmother or guardian aunt) only
   c. Both
   d. Neither
   e. Don't know
7. **What level of education did your father (or guardian uncle) complete?**
   a. No formal schooling (did not go to school)
   b. Less than primary education completed
   c. Primary education completed
   d. Junior Secondary education completed
   e. Senior Secondary education completed
   f. Tertiary education (University/Polytechnic/College of Education) completed
8. **What level of education did your mother (or guardian aunt) complete?**
   a. No formal schooling (did not go to school)
   b. Less than primary education completed
   c. Primary education completed
   d. Junior Secondary education completed
   e. Senior Secondary education completed
   f. Tertiary education (University/Polytechnic/College of Education) completed
9. **Do your parents (or guardians) live together?**
   a. Yes
   b. No

The next questions ask about your use of tobacco.

10. **Have you <u>ever tried</u> or experimented with <u>cigarette smoking</u>, even one or two puffs?**
    a. Yes
    b. No
11. **How old were you when you first tried a cigarette?**
    a. I have never tried smoking a cigarette
    b. 7 years old or younger
    c. 8 or 9 years old
    d. 10 or 11 years old
    e. 12 or 13 years old
    f. 14 or 15 years old
    g. 16 years old or older
12. **During the past 30 days, on how many days did you smoke cigarette?**
    a. 0 days
    b. 1 or 2 days
    c. 3 to 5 days
    d. 6 to 9 days
    e. 10 to 19 days
    f. 20 to 29 days
    g. All 30 days
13. **Please think about the days you <u>smoked cigarettes</u> during the past 30 days. <u>How many</u> cigarettes did you usually smoke <u>per day</u>?**
    a. I did not smoke cigarettes during the past 30 days
    b. Less than 1 cigarette per day
    c. 1 cigarette per day
    d. 2 to 5 cigarettes per day
    e. 6 to 10 cigarettes per day
    f. 11 to 20 cigarettes per day
    g. More than 20 cigarettes per day
14. **Have you <u>ever tried</u> or experimented with any form of <u>smoked tobacco</u> products <u>other than cigarettes</u> (such as pipes, cigars, water pipes/shisha, bidis)?**
    a. Yes
    b. No
15. **During the past 30 days, did you use any form of <u>smoked tobacco products other than cigarettes</u> (such as pipes, cigars, water pipes/shisha, bidis)?**
    a. Yes
    b. No
16. **Do you ever <u>smoke tobacco</u> or feel like smoking tobacco <u>first thing in the morning</u>?**
    a. I don’t smoke tobacco
    b. No, I don’t smoke tobacco or feel like smoking tobacco first thing in the morning
    c. Yes, I sometimes smoke tobacco or feel like smoking tobacco first thing in the morning
    d. Yes, I always smoke tobacco or feel like smoking tobacco first thing in the morning

17. **How soon after you <u>smoke tobacco</u> do you start to feel a strong desire to smoke again that is hard to ignore?**
    a. I don’t smoke tobacco
    b. I never feel a strong desire to smoke again after smoking tobacco
    c. Within 60 minutes
    d. 1 to 2 hours
    e. More than 2 hours to 4 hours
    f. More than 4 hours but less than one full day
    g. 1 to 3 days
    h. 4 days or more
18. **Have you <u>ever tried</u> or experimented with any form of <u>smokeless tobacco</u> products (such as snuff; chewing tobacco like tobacco leaf; applying tobacco like tobacco tooth paste or tobacco tooth powder)?**
    a. Yes
    b. No
19. **How old were you when you first tried using <u>smokeless tobacco</u>?**
    a. I have never tried using smokeless tobacco
    b. 7 years old or younger
    c. 8 or 9 years old
    d. 10 or 11 years old
    e. 12 or 13 years old
    f. 14 or 15 years old
    g. 16 years old or older
20. **During the past 30 days, did you use any form of <u>smokeless tobacco</u> products (such as snuff; chewing tobacco like tobacco leaf; applying tobacco like tobacco tooth paste or tobacco tooth powder)?**
    a. Yes
    b. No
21. **Do you ever use <u>smokeless tobacco</u> or feel like using smokeless tobacco <u>first thing in the morning</u>?**
    a. I don’t use smokeless tobacco
    b. No, I don’t use or feel like using smokeless tobacco first thing in the morning
    c. Yes, I sometimes use or feel like using smokeless tobacco first thing in the morning
    d. Yes, I always use or feel like using smokeless tobacco first thing in the morning
22. **How soon after you use <u>smokeless tobacco</u> do you start to feel a strong desire to use it again that is hard to ignore?**
    a. I don’t use smokeless tobacco
    b. I never feel a strong desire to use it again after using smokeless tobacco
    c. Within 60 minutes
    d. 1 to 2 hours
    e. More than 2 hours to 4 hours
    f. More than 4 hours but less than one full day
    g. 1 to 3 days
    h. 4 days or more

23. **Do you want to stop using <u>smokeless tobacco</u> now?**
    a. I have never used smokeless tobacco
    b. I don’t use smokeless tobacco now
    c. Yes
    d. No

The next questions ask about your feelings toward <u>stopping smoking</u>.

24. **Do you want to stop smoking now?**
    a. I have never smoked
    b. I don’t smoke now
    c. Yes
    d. No
25. **During the past 12 months, did you ever try to stop smoking?**
    a. I have never smoked
    b. I did not smoke during the past 12 months
    c. Yes
    d. No
26. **Do you think you would be able to stop smoking if you wanted to?**
    a. I have never smoked
    b. I don’t smoke now
    c. Yes
    d. No
27. **Have you ever received help or advice to help you stop smoking? (SELECT ONLY ONE RESPONSE)**
    a. I have never smoked
    b. Yes, from a program or a professional
    c. Yes, from a friend
    d. Yes, from a family member
    e. Yes, from both programs or professionals and from friends or family members
    f. No

The next questions ask about your <u>exposure to other people’s smoking</u>.

28. **During the past 7 days, on how many days has anyone smoked <u>inside your home</u>, in your presence?**
    a. 0 days
    b. 1 to 2 days
    c. 3 to 4 days
    d. 5 to 6 days
    e. 7 days

29. **During the past 7 days, on how many days has anyone smoked in your presence, <u>inside any enclosed public place</u>, other than your home (such as school, shops, restaurants, bars, shopping malls, movie theatres, airports, hospitals or clubs)?**
    a. 0 days
    b. 1 to 2 days
    c. 3 to 4 days
    d. 5 to 6 days
    e. 7 days

30. During the past 7 days, on how many days has anyone smoked in your presence, at any <u>outdoor public place</u> (such as playgrounds, sidewalks, entrance to buildings, stadium, village square, market, parks or beaches)?
   a. 0 days
   b. 1 to 2 days
   c. 3 to 4 days
   d. 5 to 6 days
   e. 7 days

31. During the past 30 days, did you see anyone smoke inside your school building or outside on school property?
   a. Yes
   b. No

32. During school hours, how often do you see teachers smoking <u>in the school building</u>?
   a. About everyday
   b. Sometimes
   c. Never
   d. Don’t know

33. During school hours, how often do you see teachers smoking <u>outdoors on school premises</u>?
   a. About everyday
   b. Sometimes
   c. Never
   d. Don’t know

34. Are you in favour of banning (disallowing) smoking <u>inside enclosed public places</u> (such as school, shops, restaurants, bars, shopping malls, movie theatres, airports, hospitals or clubs)?
   a. Yes
   b. No

35. Are you in favour of banning (disallowing) smoking at <u>outdoor public places</u> (such as playgrounds, sidewalks, entrance to buildings, stadium, village square, market, parks or beaches)?
   a. Yes
   b. No

The next questions ask about getting cigarettes.

36. The last time you smoked cigarettes during the past 30 days, how did you get them?
(SELECT ONLY ONE RESPONSE)
   a. I did not smoke any cigarettes during the past 30 days
   b. I bought them in a store or shop
   c. I bought them from a street vendor (someone selling on the street)
   d. I bought them at a kiosk (a small shop like Mallam’s shop)
   e. I got them from someone else
   f. I got them some other way

37. **During the past 30 days, did anyone refuse to sell you cigarettes because of your age?**
    a. I did not try to buy cigarettes during the past 30 days
    b. Yes, someone refused to sell me cigarettes because of my age
    c. No, my age did not keep me from buying cigarettes
38. **The last time you bought or collected cigarettes during the past 30 days, how did you buy or get them?**
    a. I did not buy cigarettes during the past 30 days
    b. I bought them in a pack
    c. I bought individual sticks (singles)
    d. I bought them in a carton
    e. I bought them in rolls
    f. I bought tobacco and rolled my own
39. **Can you buy cigarettes near your school?**
    a. Yes
    b. No
    c. I don't know
40. **On average, how much do you think a pack of 20 cigarettes costs?**
    a. 100 naira or less
    b. 101 to 150 naira
    c. 151 to 200 naira
    d. 201 to 300 naira
    e. 301 to 400 naira
    f. 401 to 500 naira
    g. More than 500 naira
    h. I don't know

The next questions ask about your knowledge of messages that are <u>against</u> using tobacco (might include cigarettes, other smoked tobacco, and smokeless tobacco).

41. **During the past 30 days, did you see or hear any media messages (adverts) against using tobacco, on television, radio, internet, billboards, posters, newspapers, magazines, or movies?**
    a. Yes
    b. No
42. **During the past 30 days, did you see or hear any messages against using tobacco, at sports events, fairs, concerts, community events, or religious or social gatherings?**
    a. I did not go to sports events, fairs, concerts, community events, or social gatherings in the past 30 days
    b. Yes
    c. No
43. **During the past 30 days, did you see any health warnings on cigarette packages?**
    a. I did not see any cigarette packages
    b. Yes, but I didn't think much of them
    c. Yes, and they led me to think about quitting smoking or not starting smoking
    d. No

44. **Has anyone in your family discussed the harmful effects of smoking tobacco with you?**
    a. Yes
    b. No
45. **During the past 12 months, were you taught in any of your classes about the dangers of tobacco use?**
    a. Yes
    b. No
    c. I don't know
46. **During the past 12 months, did you read in your school texts or books about the health effects of tobacco?**
    a. Yes
    b. No
    c. I do not have school texts or books
47. **During the past 12 months, did you discuss in any of your classes the reasons why people your age use tobacco?**
    a. Yes
    b. No
    c. Not sure

The next questions ask about your <u>knowledge of advertisements or promotions</u> for tobacco (might include cigarettes, other smoked tobacco, and smokeless tobacco).

48. **During the past 30 days, did you see any people using tobacco when you watched TV, videos or movies?**
    a. I did not watch TV, videos or movies in the past 30 days
    b. Yes
    c. No
49. **During the past 30 days, did you see any advertisements or promotions for tobacco products at points of sale (such as stores, shops, kiosks, etc)?**
    a. I did not visit any points of sale in the past 30 days
    b. Yes
    c. No
50. **Would you ever use or wear something that has a tobacco company name (like Benson and Hedges, Rothmans, Dunhill, etc) or picture on it, such as a lighter, t-shirt, hat, or sunglasses?**
    a. Yes
    b. Maybe
    c. No
51. **Do you have something (for example, t-shirt, pen, or backpack) with a tobacco product brand on it like Benson and Hedges, Rothmans, Dunhill etc?**
    a. Yes
    b. No
52. **Has a person working for a tobacco company ever offered you a free tobacco product?**
    a. Yes
    b. No

The next questions ask about your attitudes and beliefs about using tobacco.

53. Do your parents (or guardians) smoke tobacco?
   a. None
   b. Both
   c. Father (or guardian uncle) only
   d. Mother (or guardian aunt) only
   e. Don't know

54. Do any of your closest friends smoke tobacco?
   a. None of them
   b. Some of them
   c. Most of them
   d. All of them

55. About how many students in your grade/form smoke tobacco?
   a. None of them
   b. Some of them
   c. Most of them
   d. All of them

56. If one of your best friends offered you a tobacco product, would you use it?
   a. Of course not
   b. Maybe not
   c. Maybe yes
   d. Of course yes

57. At anytime during the next 12 months, do you think you will use any form of tobacco?
   a. Of course not
   b. Maybe not
   c. Maybe yes
   d. Of course yes

58. **Once someone has started smoking tobacco, do you think it would be difficult for them to quit (or give up)?**
   a. Of course not
   b. Maybe not
   c. Maybe yes
   d. Of course yes

59. Do you think smoking tobacco helps people feel more relaxed or less relaxed at celebrations, parties, or in other social gatherings?
   a. More relaxed
   b. No difference whether smoking or not
   c. Less relaxed

60. Do you think young people who smoke tobacco have more or less friends?
   a. More friends
   b. No difference from non-smokers
   c. Less friends

61. Do you think smoking tobacco makes young people look more or less attractive?
   a. More attractive
   b. No difference from non-smokers
   c. Less attractive

62. Do you think smoking tobacco is harmful to your health?
   a. Of course not
   b. Maybe not
   c. Maybe yes
   d. Of course yes

63. Do you think the smoke from other people's tobacco smoking is harmful to you?
   a. Of course not
   b. Maybe not
   c. Maybe yes
   d. Of course yes

64. Do you think it is safe to smoke tobacco for only a year or two as long as you quit (or give up) after that?
   a. Of course not
   b. Maybe not
   c. Maybe yes
   d. Of course yes

65. Do you think the sale of tobacco products to minors (< 18 years) should be banned?
   a. Yes
   b. No

66. Do you agree or disagree with the following: "I think I might enjoy smoking a cigarette."
   a. I currently smoke cigarettes
   b. Strongly agree
   c. Agree
   d. Disagree
   e. Strongly disagree

Thank you for participating in the survey!
